# Fracture severity dependence of bone and muscle performance in patients following single or multiple vertebral fractures

**DOI:** 10.3389/fendo.2024.1423650

**Published:** 2024-11-06

**Authors:** Chenggui Zhang, Yang Li, Guodong Wang, Jianmin Sun

**Affiliations:** Department of Orthopedics, Shandong Provincial Hospital Affiliated to Shandong First Medical University, Jinan, Shandong, China

**Keywords:** osteoporosis, vertebral fracture, bone, muscle, multiple fractures

## Abstract

**Background:**

Few studies focus on the clinical, laboratory, radiological, and biological characteristics of bone and muscle of multiple vertebral fractures, which are associated with a more poor prognosis compared with single fracture.

**Purpose:**

To compare the BMD, bone turnover, muscularity, fatty infiltration of muscle, and prevalence of co-morbidities in patients with single and multiple vertebral fractures.

**Methods:**

We recruited 100 patients with single fracture (age 66.96 ± 8.24 years) and 100 with multiple fractures (age 69.90 ± 7.80 years); performed dual-energy X-ray absorptiometry of the femoral neck, hip, and lumbar vertebrae; and measured biochemical markers of bone turnover, muscularity, and fatty infiltration.

**Results:**

Patients with multiple vertebral fractures had lower hip BMD (*p*=0.010) than those with single fractures, but there was no difference in femoral neck and lumbar vertebral BMD nor in muscularity. However, fatty infiltration, an indicator of muscle quality, was significantly higher in participants with multiple fractures (*p*=0.006). Diabetes was significantly more common in patients with multiple fractures (*p*=0.042). There were no significant differences in markers of bone turnover, and Seperman analyses showed no correlations of CTX-1 or tPINP with the BMD of the hip, femoral neck, or lumbar spine. However, high CTX-1 was associated with high tPINP (r=0.4805; *p*<0.0001), and marked fatty infiltration was associated with low hip, lumbar vertebral, and femoral neck BMD. Cox regression analyses showed that age (OR 1.057; 95% CI 1.016–1.101; *p*=0.006) and low hip BMD (OR 0.016; 95% CI, 0.000–0.549; *p*=0.022) were associated with a higher risk of multiple fractures.

**Conclusion:**

Patients with multiple fractures tend to have lower hip BMD, a history of type 2 diabetes, and more substantial fatty infiltration of muscle than in those with single fractures. Age and hip BMD rather than lumbar vertebrae BMD were found to be independent risk factors for multiple vertebral compression fractures, implying that hip BMD may be a more sensitive predictor for multiple vertebral fractures. More improvements in hip BMD and focus on older persons may be useful means of preventing multiple fractures.

## Introduction

With the rapid aging of the population, the incidence of osteoporotic fractures is predicted to increase markedly, which is likely to result in an increase in mortality, a loss of quality-adjusted lifespan, and higher care costs, all of which will place substantial burdens on individual patients and societies (1). Of all the types of osteoporotic fractures, vertebral compression fractures are considered to be the most common, with an estimated 1.4 million new clinically relevant vertebral fractures occurring worldwide each year (2, 3). Vertebral compression fractures often lead to severe chronic back pain, disability, a reduction in quality of life, and spinal kyphosis (4).

Furthermore, patients who sustain an initial osteoporotic vertebral fracture are at higher risk of further fractures, a phenomenon that is referred to as a vertebral fracture cascade (5, 6). It has been reported that there is a 2–10-fold higher risk of fracture following an initial fracture and that the 5-year risk of subsequent fracture is relatively high (7, 8). In particular, there is a high risk of osteoporotic fracture within 1–2 years of the initial fracture, when patients are defined as being at “imminent fracture risk” (9). In addition, the risk of imminent fracture is particularly high in older patients and perhaps also in those with an index vertebral fracture (10). Imminent fracture has been shown to be better predicted by a vertebral fracture than by any other type of major osteoporotic fracture (11).

Multiple vertebral compression fractures are common in the clinical setting and are usually associated with more severe clinical outcomes, including intractable back pain and immobilization-related co-morbidities, including akinesia, deep venous thrombosis, pulmonary infection, and cardiovascular and cerebrovascular events (12, 13). Multiple vertebral fractures that occur within a short period of time, and in particular, a cluster of vertebral compression fractures, can result in serious problems (14). However, although there have been many studies of the clinical, laboratory, radiological, and biological characteristics of bone and muscle associated with single vertebral fractures (6, 15), there have been few studies focusing on multiple vertebral fractures, which are associated with a poor prognosis.

Therefore, in the present study, we aimed to quantify the bone mineral density (BMD), bone metabolism, and muscle-related parameters associated with multiple vertebral fractures, and compare their values in patients with single or multiple vertebral fractures. In this way, we aimed to identify risk factors that would help predict or prevent multiple vertebral compression fractures and potentially indicate useful interventions.

## Materials and methods

### Study sample

We performed a retrospective case-control study that was approved by the Ethics Committee of The First Affiliated Hospital of Shan Dong Medical University. We enrolled 200 patients aged >55 years who had experienced vertebral compression fracture between January 2019 and December 2021. Low-energy fractures occurring in elderly female patients aged 55 and above were defined as osteoporotic fractures based on the previous research (16). Patients who had experienced two or more new vertebral compression fractures at the time of admission were defined as having multiple fractures, and those who had one vertebral compression fracture were defined as having a single fracture.

**Figure 1:**
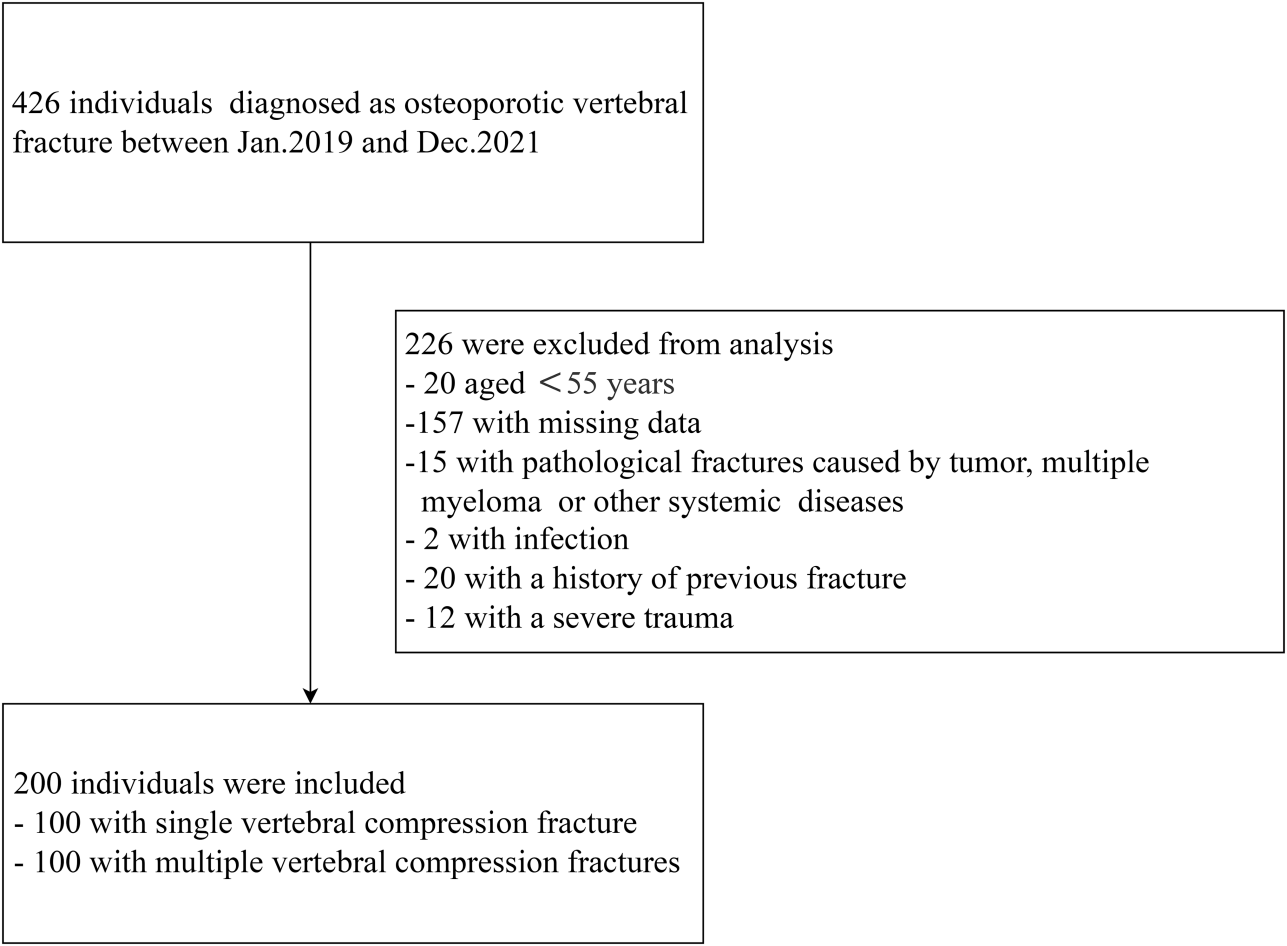
Flow diagram of the study sample.

All the patients for whom preoperative magnetic resonance imaging (MRI) confirmed the presence of an osteoporotic vertebral fracture and fat suppression imaging showed a high signal intensity in the fractured vertebral body were considered for inclusion in the study. Individuals were excluded if they had (1) acute vertebral compression fracture caused by severe trauma, such as a car accident or a fall; (2) a primary or metastatic tumor; (3) multiple myeloma or another systemic disease; (4) infection; or (5) an incomplete set of data, including a lack of BMD or bone metabolic index data (**Figure 1**).

### Data collection

Clinical and pharmacological data were collected for each participant during an interview. The following information was collected for each participant: basic demographic data (sex, age, height, and body mass), preoperative absolute BMD, bone metabolism parameters, the number and location of vertebral body fractures, fracture history prior to the index fracture, the circulating hemoglobin and cholesterol concentrations, and the presence of chronic diseases (hypertension, diabetes, coronary atherosclerosis, or chronic obstructive pulmonary disease).

The BMDs of lumbar vertebrae 1–4 and the femoral neck and hip region were measured using dual energy X-ray absorptiometry (DEXA). For patients who had undergone percutaneous vertebroplasty and pedicle screw fixation, we avoid the areas with bone cement and fixation during X-ray examination to accurately reflect the patients’ bone density. The serum β-isomerized C-terminal telopeptide (β-CTX), total procollagen type I propeptide (tPINP), parathyroid hormone (PTH), 25-hydroxyvitamin D (25OHD), and osteocalcin concentrations were measured using radioimmunoassays and enzyme-linked immunoassays.

MRI was used to quantify the degrees of fatty infiltration and atrophy of the paraspinal muscles. Lumbar muscularity was calculated using the muscle-VB CSA ratio to reduce the bias caused by body size and disk pathology, as previously described (17). A pseudocoloring technique was used to measure the percentage fat infiltration, as previously described (18). Briefly, using the pseudocoloring tool of the program, the bright pixels of the adipose tissue in the MR images were colored red and the percentage of the total muscle compartment area that was red was calculated.

### Statistical analysis

Continuous data are expressed as means ± standard deviations, and the independent samples *t*-test (continuous data, normal distribution) or Mann-Whitney U test (continuous data, abnormal distribution) was used to compare the groups. Categorical data are presented as counts and percentages and were analyzed using the chi-square test. Correlations were evaluated using Spearman analysis when at least one categorical variable was involved and Pearson analysis for continuous data. Binary logistic regression analysis was used to identify potential risk factors for multiple fractures. The results are reported as adjusted hazard ratios (HRs) and 95% confidence intervals (95% CIs). Statistical analyses were performed using SPSS 17.0 statistical software (SPSS Inc., Chicago, IL, USA) and *P* < 0.05 was considered to represent statistical significance.

## Results

We recruited 100 patients with single vertebral compression fracture and 100 patients with multiple vertebral compression fractures for comparison. **Table 1** shows the characteristics of the participants. The participants with multiple fractures were older (multiple fractures *vs*. single fracture: 69.90 ± 7.80 years *vs*. 66.96 ± 8.24 years, respectively; *P*=0.010) than those with single vertebral fracture. There were no significant differences in the height or body mass of the participants with single or multiple vertebral fractures. There were no significant differences between groups with respect to the prevalences of co-morbidities, such as coronary artery disease and hypertension. However, patients with multiple fractures were more likely to have type 2 diabetes (24% *vs*. 12%, *P*=0.042) than those with a single fracture. The prevalences of bisphosphonate use and steroid treatment prior to admission did not differ between participants with single or multiple vertebral fractures (**Table 1**).

**Table 1:** General characteristics of the study sample.

	Single fracture (N=100)	Multiple fractures (N=100)	P-value
Age (years)	66.96±8.24	69.90±7.80	**0.010**
Male (n)	12	14	0.834
Female (n)	88	86	
Weight (kg)	68.46±10.47	66.16±8.31	0.088
Height (cm)	168.32±7.03	167.2±6.69	0.250
Smoking (n)	12	15	0.680
Alcohol (n)	10	14	0.515
Hypertension (n)	9	11	0.487
Type II diabetes (n)	12	24	**0.042**
Coronary artery disease (n)	13	16	0.424
History of steroid use (n)	15	21	0.203
Bisphophonate use (n)	12	6	0.329

Data are expressed as mean ± SD or number. Significant values are shown in bold. *P*-values were calculated using the chi-square test for categorical data and the independent samples *t*-test for continuous data.

Most of the vertebral fractures were at the thoracolumbar junction (T12–L1), with at least one vertebral compression fracture in T12 or L1 comprising 57% of all the fractured vertebrae in the multiple fractures group and 42.5% of all the fractured vertebrae in the single fracture group (**Table 2**).

**Table 2:** Distribution of osteoporotic vertebral compression fractures.

	Single fracture		Multiple fractures	
	N	Incidence (%)	N	Incidence (%)
T2	0	0	0	0
T3	0	0	0	0
T4	1	1	0	0
T5	1	1	1	0.53
T6	0	0	1	0.53
T7	2	2	1	0.53
T8	2	2	8	4.3
T9	1	1	6	3.2
T10	4	4	8	4.3
T11	8	8	16	8.6
T12	30	30	35	18.8
L1	27	27	44	23.7
L2	14	14	24	12.9
L3	5	5	21	11.3
L4	4	4	13	6.99
L5	1	1	8	4.3

**Figure 2:**
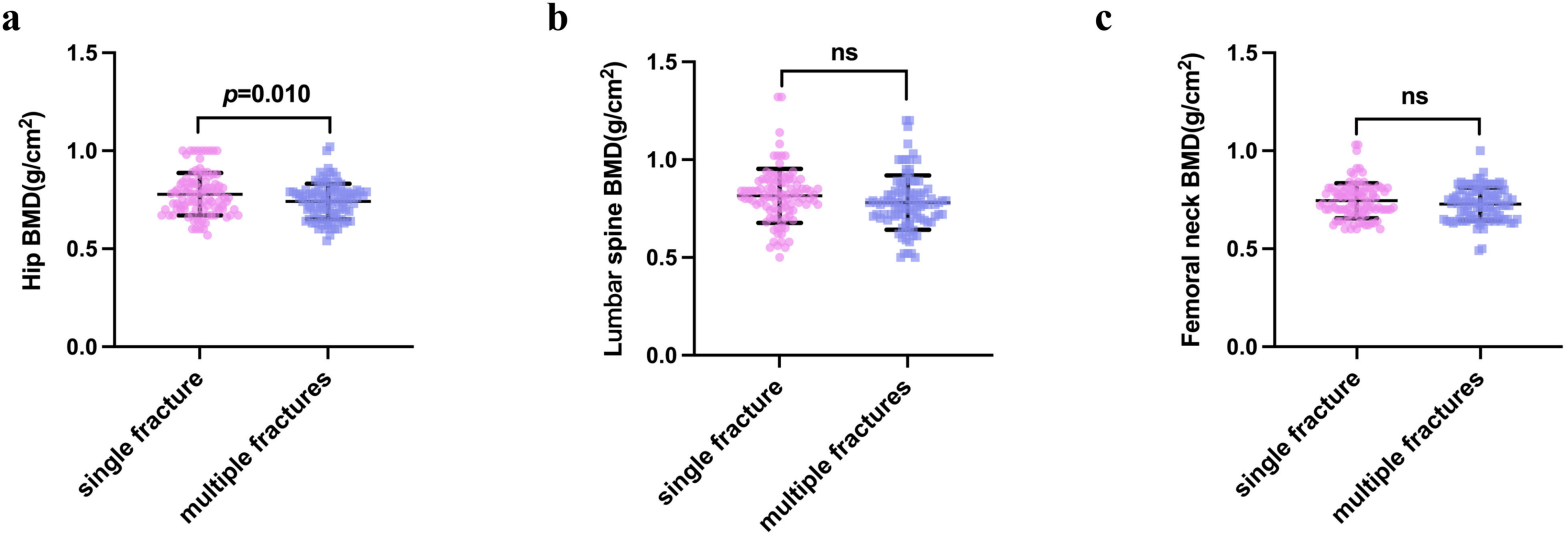
Bone mineral density of the hip **(A)**, femoral neck **(B)**, and lumbar vertebrae **(C)** in the single vertebral fracture and multiple vertebral fracture groups. *P*-values were calculated using the independent samples *t*-test. NS indicates that P > 0.05, which means there is no statistical significance between the groups.

Participants with multiple vertebral compression fractures tended to have a lower BMD. There was significantly lower BMD of the hip region in the multiple fracture group than in the single fracture group (*P*=0.010) (**Table 2** and **Figure 2A**). The lumbar BMD tended to be lower in the multiple fractures group, but there was no significant difference between this and the single vertebral compression fracture group (*P*=0.068). There was no difference in the BMD of the femoral neck between the two groups (*P*=0.173) (**Table 2** and **Figures 2B, C**).

We found no significant difference in the concentration of β-CTX (a marker of osteoclastic function) between participants with multiple fractures and those with a single fracture (*P*=0.384). However, the concentration of tPINP (a marker of osteoblastic function) tended to be higher in participants with multiple fractures in (*P*=0.083). The concentrations of PTH, VitD-T, 25-(OH)2D3, and osteocalcin did not differ between the groups (**Table 3**).

**Table 3:** Comparison of indices of bone turnover and DEXA data between the groups.

	Single fracture	Multiple fractures	P-value
Lumbar spine BMD (g/cm^2^)	0.82±0.14	0.78±0.14	**0.068**
Femoral neck BMD (g/cm^2^)	0.74±0.09	0.73±0.08	0.173
Total hip BMD (g/cm^2^)	0.78±0.11	0.74±0.09	**0.010**
tP1NP (ng/mL)	68.9±31.41	77.2±35.98	0.083
β-CTX (ng/mL)	0.73±0.34	0.77±0.31	0.384
PTH (ng/L)	39.10±21.12	40.24±22.41	0.712
25OHD (μg/L)	15.50±8.98	17.24±9.87	0.192
OST	18.42±9.75	19.03±8.18	0.634

Data are expressed as mean ± SD. Significant values are shown in bold. *P*-values were calculated using the independent samples *t*-test.

**Figure 3:**
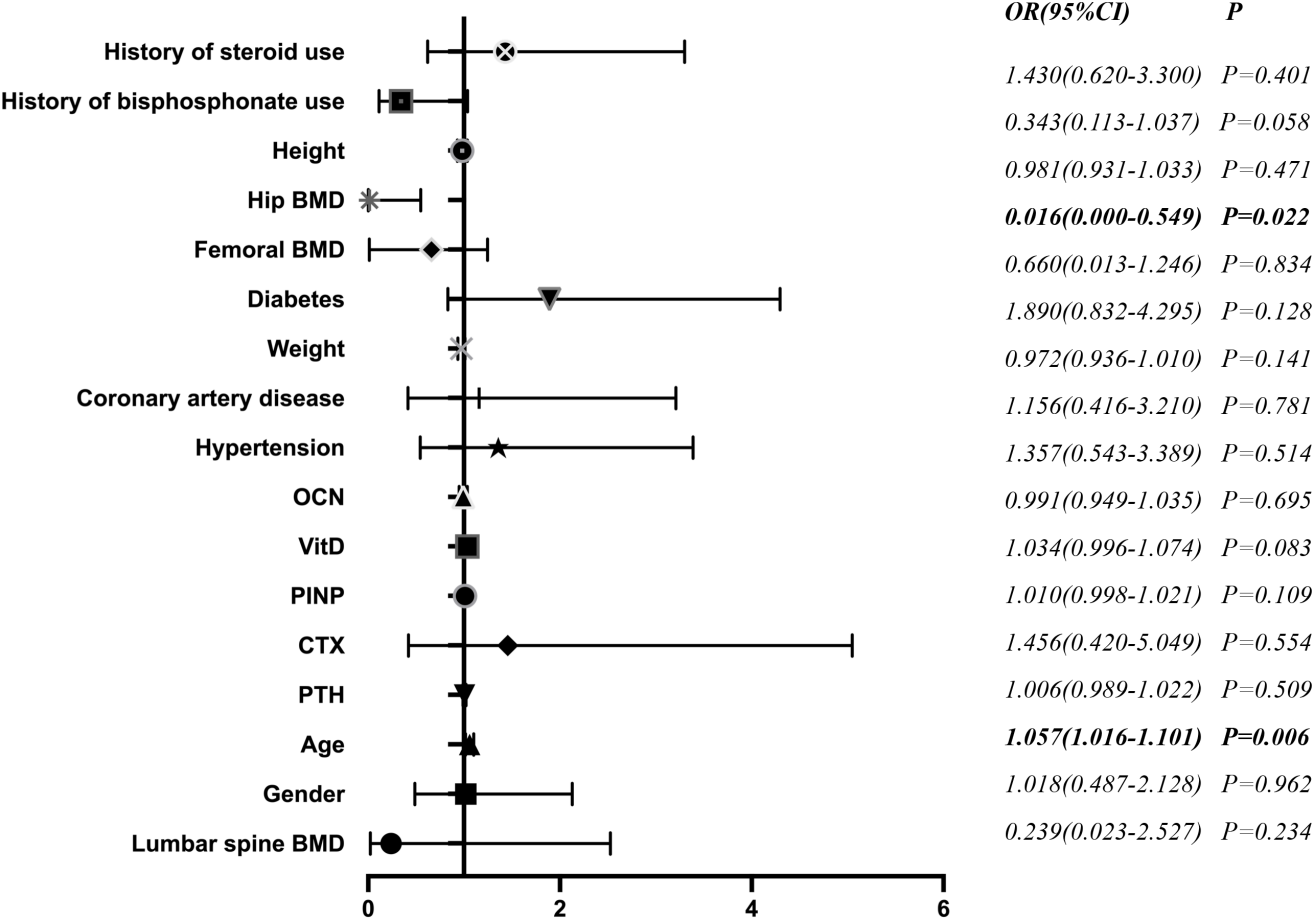
Relationships of CTX and tPINP with the BMD of the lumbar vertebrae and femoral neck Correlations of the serum CTX and tPINP concentrations with the BMD of the lumbar spine and femoral neck are shown. BMD, bone mineral density; β-CTX, β-isomerized C-terminal telopeptides; tPINP, total procollagen type I propeptides.

We next aimed to identify independent risk factors for multiple vertebral compression fractures. The BMD of the hip region was significantly higher in the multiple vertebral compression fracture group. The participants in the multiple vertebral compression fracture group were older than those in the single fracture group. In addition, type 2 diabetes was more prevalent in the multiple fracture group. We used these findings to identify independent risk factors, including the age, prevalence of type 2 diabetes, and BMD of the hip region in multivariate logistic regression. We found that age (odds ratio (OR) 1.057; 95% CI 1.016–1.101; *P*=0.006) and low hip BMD (OR 0.016; 95% CI 0.000–0.549; *P*=0.022) were independent risk factors for multiple vertebral compression fracture in the multivariate analysis, whereas type 2 diabetes (*P=*0.128) was not (**Figure 3**).

**Figure 4:**
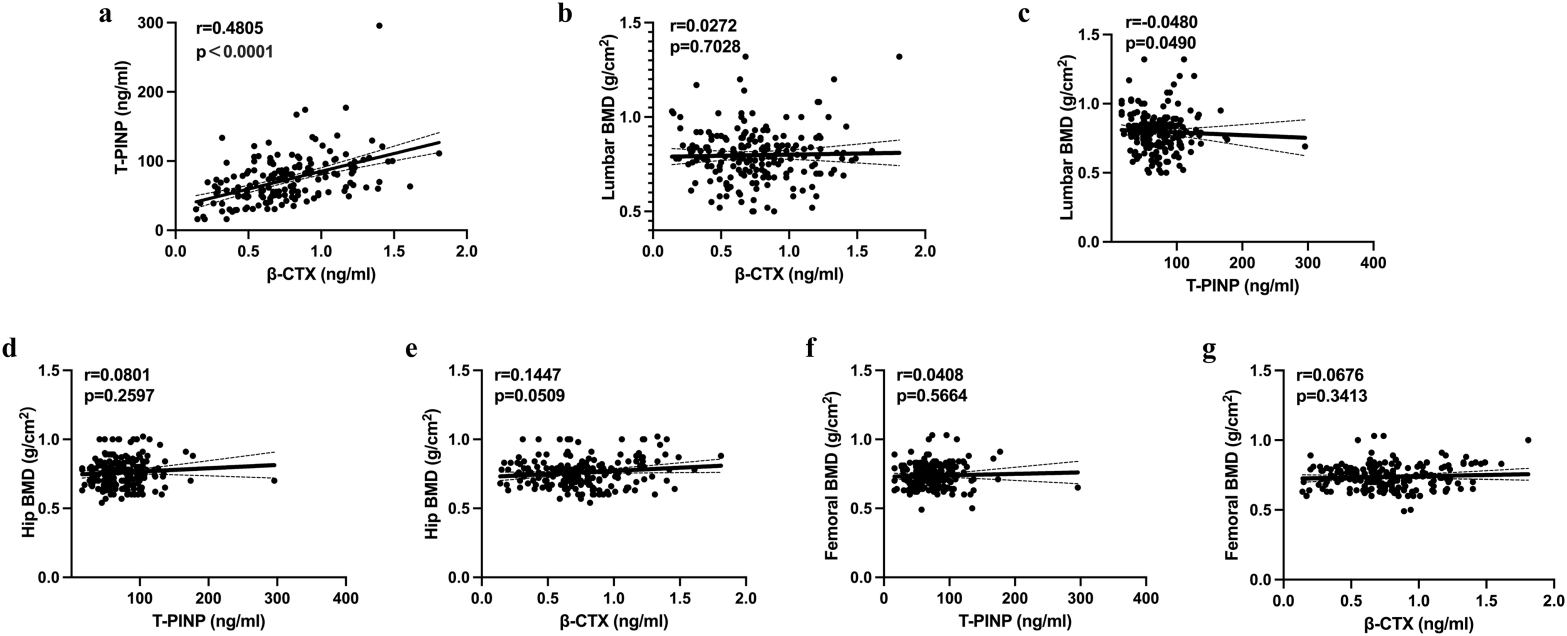
Results of the binary logistic regression analysis to identify factors potentially influencing the risk of multiple fractures **(A–G)**. BMD, bone mineral density; β-CTX, β-isomerized C-terminal telopeptides; tPINP, total procollagen type I propeptides; OCN, osteocalcin; VitD, vitamin D; PTH, parathyroid hormone.

There was a significant relationship between the β-CTX and tPINP concentrations (r=0.4805; *P*<0.0001) (**Figure 4A**), but there were no other significant relationships among the other bone metabolism parameters. We also evaluated the relationships between the BMDs at various locations and the indices of bone metabolism, and found no significant relationships across the entire cohort (**Figures 4B–G**).

**Figure 5:**
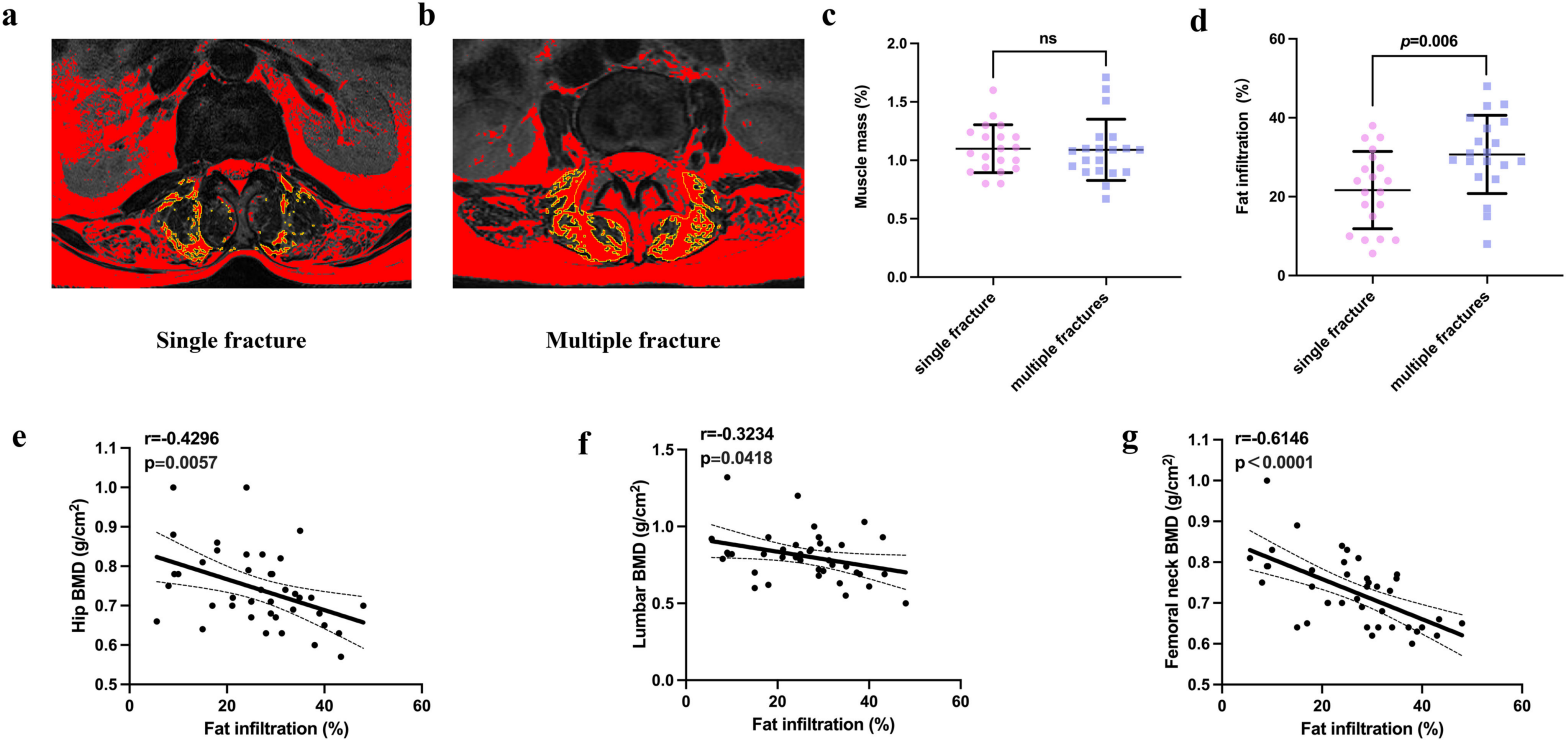
Comparison of the muscle mass and fatty infiltration of participants in the single and multiple fracture groups **(A, B)**. Representative graphs of the fatty infiltration of muscle in participants in the single and multiple fracture groups. **(C, D)**. Quantification of muscle mass and fatty infiltration in the two groups. **(E–G)**. Correlation analysis of the relationships of fatty infiltration with the bone mineral density of the hip, lumbar vertebrae, and femoral neck. NS indicates that P > 0.05, which means there is no statistical significance between the groups.

Fatty infiltration of the back muscle was more prevalent in participants with multiple fractures. Although the muscularity of the two groups did not significantly differ, participants with multiple vertebral compression fractures showed more fatty change in their back muscles at the L4/5 level than those with single fractures (*P*=0.006). (**Figures 5A–D**). In addition, fatty infiltration of the back muscle at the L4/5 level correlated with the BMD of the hip region (r=0.4296; *P*=0.0057), lumbar vertebrae (r=0.3234; *P*<0.0418), and femoral neck (r=0.6146; *P*<0.0001) across the entire cohort (**Figures 5E–G**).

## Discussion

The present findings show that people with multiple vertebral compression fractures have a lower BMD of the hip region than those with single fractures, but that there are no differences in the BMDs of the lumbar vertebrae and femoral neck. In addition, bone metabolism parameters did not differ between these groups, nor did they correlate with the BMDs of the hip, femoral neck, or lumbar vertebrae. However, there was a correlation between the concentrations of the bone formation marker t-P1NP and the bone resorption marker CTX-I. We also found that the age, BMD of the hip region, and prevalence of co-morbid diabetes significantly differed between the groups, and that age and the BMD of the hip region are independent risk factors for multiple fractures. Finally, we found that fatty infiltration of the back muscle is more severe in individuals with multiple compression fractures and correlates with the BMD of the hip region.

To date, there have been no studies of the relationship between BMD and bone turnover parameters in patients with single or multiple vertebral compression fractures. However, patients who develop a vertebral compression fracture are at substantial risk of additional fractures (11). In our previous study, we found that multiple fractures in mice are associated with more severe bone loss than single fractures, which may imply a higher risk of further fractures in individuals with multiple fractures than in those with single fractures (19). Interestingly, in the present study, we found that the BMD of the hip region differed between the groups, while that of the lumbar vertebrae did not. Similarly, a recent study showed that a change in lumbar vertebral BMD is not closely associated with a reduction in vertebral fracture risk and that the changes in the BMDs of both the hip region and femoral neck specifically may explain a larger proportion of the reduction in fracture risk than lumbar vertebral BMD (20). Another study also showed that treatment-related increases in total hip BMD are associated with a lower risk of major osteoporotic fracture, including of the hip and clinical vertebral fracture, than a stable BMD, and that decreases in BMD are associated with higher fracture risk. In addition, changes in vertebral BMD were found not to be independently associated with fracture risk (21). The present findings also imply that hip BMD is a better indicator of the risk of multiple fractures than vertebral BMD.

Bone turnover markers (BTMs) are circulating biochemical substances that reflect *in vivo* bone formation and resorptive activity. tPINP is a marker of bone formation and β-CTX reflects osteoclast activity, and therefore serves as a marker of bone resorption (22). Previous studies have shown that the serum concentrations of BTMs, including tPINP and β-CTX, are high following a fracture (23). In the present study, the concentrations of both tPINP and β-CTX were high in both the single and multiple vertebral compression fracture groups. In addition, the tPINP and β-CTX concentrations were significantly correlated, which is consistent with high bone turnover following fracture. This is similar to the findings of a previous study that “transient osteoporosis” may occur during the regional acceleratory phenomenon because of the time lag between resorption and formation that characterizes bone healing (24).

In fact, the use of BTMs to predict fracture risk remains controversial. It has been reported that the serum CTX and tPINP concentrations may not be suitable for the prediction of hip fracture risk (25). However, other previous studies have shown that high BTM concentrations may predict fracture risk independently of BMD (26), and that early BTM changes are associated with subsequent changes in BMD following the treatment of osteoporosis (27). In the present study, the concentrations of BTMs, including β-CTX and tPINP, did not differ between the single and multiple vertebral compression fracture groups and did not all correlate with hip BMD. Therefore, it may be that the increases in BTM concentrations caused by fracture are not paralleled by similar changes in BTMs in patients with osteoporosis but no fracture, making them less suitable for use in predicting fractures or estimating the magnitude of the risk.

Age is associated with decreases in both physical abilities and general health (28), and bone loss and structural damage are key negative outcomes of advancing age (29). In the present study, the participants in the multiple vertebral compression fracture group were older than those in the single fracture group. In addition, increasing age was found to be an independent risk factor for multiple vertebral compression fractures. This is consistent with previous findings that advanced age is a potent risk factor for fracture at nearly all skeletal sites and that the risk increases markedly with age (29, 30).

In the present study, type 2 diabetes was more common in the multiple vertebral compression fracture group, whereas the prevalences of hypertension and coronary artery disease did not differ between the groups. However, type 2 diabetes was found not to be an independent risk factor for multiple fractures. Multiple previous large-scale prospective studies and meta-analyses have shown that type 2 diabetes is associated with non-vertebral fracture risk, and particularly with hip fracture risk (31–33). However, there is no conclusive evidence concerning vertebral fracture risk in individuals with type 2 diabetes, because previous studies have shown low-to-no association or greater risk (34–36). The relationship between diabetes and bone is in general intriguing. In spite of having higher levels of areal BMD, assessed using DEXA, patients with type 2 diabetes sustain larger numbers of fractures than those without. This may be at least in part explained by alterations in the bone microarchitecture, including impairments in bone material properties and increases in cortical porosity, two key skeletal abnormalities that contribute to skeletal fragility in patients with type 2 diabetes (32, 37). Therefore, a comparison of the bone microarchitecture, in addition to the BMD, of patients with multiple vertebral compression fracture or single fracture is also of great value.

In the present study, the majority of vertebral fractures occurred at the thoracolumbar junction (T12–L1 vertebrae) in both the single and multiple vertebral compression fracture groups. These data are consistent with previous findings that vertebral fractures occur more frequently in the thoracolumbar region of the vertebral column because of the higher load-bearing in this region than in the thoracic region, owing to the absence of support from the ribcage (6, 38).

Numerous previous studies have shown that there are close functional and developmental relationships between muscle and bone mass (39–41). These links are not exclusively mechanical, but are also mediated through complex bidirectional crosstalk, involving osteokines and myokines (42, 43).For example, bone can secrete Prostaglandin E2 (PGE2), Wnt3a and other cytokines that act on muscle, while muscle can secrete insulin-like growth factor-1 (IGF-1), basic fibroblast growth factor (FGF-2), interleukin-6 (IL-6), and other myokines that influence bone (39, 44). In the present study, although the lumbar vertebral BMDs and total muscle masses of the single and multiple vertebral compression fracture groups did not differ significantly, fatty infiltration of the back muscle, which reflects muscle quality, was more severe in the multiple vertebral compression fracture group and significantly correlated with lumbar vertebral and hip BMD. These findings are consistent with those of a previous study showing that the BMDs of the lumbar vertebrae, hip region, femoral neck, and whole body were positively associated with appendicular lean mass index and negatively associated with fat mass index (45). Furthermore, fatty infiltration of the paraspinal muscle increased as lumbar BMD decreased, even after adjustment for sex, age, and BMI (46). A previous study also showed more substantial effects of local interactions than systemic interactions between muscle and bone, and specifically that appendicular skeletal muscle mass is more closely associated with femoral neck BMD than with lumbar vertebral BMD or trabecular bone score (47). In addition, the BMD of the lower lumbar vertebrae seems to be independently associated with fatty infiltration of the paraspinal muscle (48). The greater fatty infiltration identified in the multiple vertebral compression fracture group *versus* the single fracture group may be explained by older patients with lower muscle quality tending to experience a larger number of vertebral compression fractures over a short period of time.

The present study had a number of strengths. Notably, to the best of our knowledge, previous studies have not comprehensively compared the key clinical characteristics of patients with single or multiple vertebral compression fractures, including their BMD, bone metabolism, muscle parameters, and the presence of co-morbidities. Importantly, we have shown that hip BMD, but not lumbar vertebral BMD, is lower in patients with multiple vertebral compression fractures. This finding implies that hip BMD may be a more useful index for the prediction of multiple fractures. In addition, we were able identify age and hip BMD as independent risk factors for multiple vertebral compression fractures. This lends further support to the idea that improvements in hip BMD and focusing on the care of older persons may be useful for the prevention of multiple fractures. Finally, the results confirm that fatty infiltration of back muscle is more severe in patients with multiple vertebral compression fracture and correlates with the BMDs of the hip and lumbar vertebrae, which is further evidence of the close relationship between bone and muscle. Nevertheless, the study had several limitations. Firstly, we did not study the changes in hip and lumbar vertebral BMD following fracture, just the BMD within 1 month of the index fracture. Therefore, we could not investigate the longitudinal relationship between BMD and multiple fractures. However, the occurrence of an initial fracture should be regarded as an opportunity to identify patients at risk and put interventions in place to prevent a second fracture. Secondly, we excluded patients with multiple fractures that included one or more older fractures. Therefore, the results may not be applicable to this situation and further studies are needed to investigate the factors influencing the occurrence of multiple fractures, including older fractures. Thirdly, mortality data and follow-up data were not obtained in the research. Future studies with larger sample size or the use of public database may better capture differences in mortality and explore the long-term impact of fractures on bone health, which would be of significant importance.

## Conclusion

We have shown that low hip BMD, a history of type 2 diabetes, and substantial fatty infiltration of muscle are more common in patients with multiple fractures than in those with single fractures. In addition, age and hip BMD rather than lumbar vertebrae BMD were found to be independent risk factors for multiple vertebral compression fractures. More improvements in hip BMD and focus on older persons may be useful means of preventing multiple fractures.

## Data Availability

The raw data supporting the conclusions of this article will be made available by the authors, without undue reservation.
